# C-Kit, a Double-Edged Sword in Liver Regeneration and Diseases

**DOI:** 10.3389/fgene.2021.598855

**Published:** 2021-02-02

**Authors:** Weina Wang, Liyan Shui, Yanning Liu, Min Zheng

**Affiliations:** State Key Laboratory for Diagnosis and Treatment of Infectious Diseases, National Clinical Research Center for Infectious Diseases, Collaborative Innovation Center for Diagnosis and Treatment of Infectious Diseases, The First Affiliated Hospital, College of Medicine, Zhejiang University, Hangzhou, China

**Keywords:** c-kit, liver regeneration, stem cell, liver disease, HCC

## Abstract

Previous studies have reported an important role of c-kit in embryogenesis and adulthood. Activation of the SCF/KIT signal transduction pathway is customarily linked to cell proliferation, migration and survival thus influence hematopoiesis, pigmentation, and spermatogenesis. The role of c-kit in the liver is controversial, it is however argued that it is a double-edged sword in liver regeneration and diseases. First, liver c-kit^+^ cells, including oval cells, bile epithelial cells, and part of hepatocytes, participate in liver tissue repair by regenerating target cells according to the type of liver injury. At the same time, c-kit^+^ mast cells, act as immature progenitors in circulation, playing a critical role in liver fibrosis. Furthermore, c-kit is also a proto-oncogene. Notably, c-kit overexpression regulates gastrointestinal stromal tumors. Various studies have explored on c-kit and hepatocellular carcinoma, nevertheless, the intricate roles of c-kit in the liver are largely understudied. Herein, we extensively summarize previous studies geared toward providing hints for future clinical and basic research.

## Introduction

C-kit also known as CD117, is a type III receptor tyrosine kinase coded by the KIT gene. It has a stem cell growth factor (SCF) as its ligand, hence referred to as the stem cell growth factor receptor (SCFR) (Fujio et al., 1996). C-kit is encoded by white spotting (W) locus with more than 30 mutations while SCF is encoded by steel (Sl) locus also with natural mutations (Lev et al., 1994). In mice, total loss of c-kit or SCF function causes embryonic lethal, however, mice with one of these mutations (W or Sl mice) can survive.

Previous studies have demonstrated the fundamental role of c-kit in embryogenesis and adulthood. C-kit can be detected in several normal cells including, hematopoietic stem cells (HSCs), oval cells, intestinal cells of Cajal, germ cells, melanocytes among others. Activation of the SCF/c-kit signal transduction pathway is usually linked to cell proliferation, migration, and survival, and thus regulate crucial functions in hematopoiesis, pigmentation, and spermatogenesis (Besmer et al., 1993). The liver is the only visceral organ that possesses the capacity to regenerate. As long as 30–35% of the liver remains, the remnant liver will undergo hyperplasia until the normal liver is re-established. Approximately 20% of hepatocytes located mainly at the periportal and pericentral regions express c-kit located with a weak staining intensity participates in liver regeneration (Yushkov et al., 2011). Hepatic resident stem cells and bone marrow-derived stem cells (BMSCs), both of which express c-kit, can differentiate into various cells according to the specific injuries. Moreover, transplantation of c-kit positive (c-kit^+^) oval cells, and BMSCs is a potential factor of improving liver regeneration.

Under pathological conditions, excessive c-kit signaling is often associated with tumor formation and progression in gastrointestinal stromal tumors (GISTs), melanoma, small cell lung carcinoma, testicular carcinoma, etc. Imatinib, a relatively selective tyrosine inhibitor, is a target particularly of BCR-ABL, platelet-derived growth factor receptor (PDGFR), and c-kit. Imatinib is commonly used in the treatment of GIST. Piebaldism is a rare autosomal dominant disorder in which interstitial cells of Cajal are lost due to the loss-of-function mutation of c-kit (Giebel and Spritz, 1991). In liver, c-kit^+^ oval cells malignant transformation might be one of the possible mechanisms of hepatocarcinogenesis (Fang et al., 2004). Moreover, c-kit mutation or overexpression has been reported in hepatocellular carcinomas (HCC) (Potti et al., 2003; Yan et al., 2018). Besides, c-kit^+^ mast cells play a key role in fibrogenesis, specifically in cholestatic/biliary diseases associated with fibrosis.

Nonetheless, the complex roles of c-kit and liver are largely understudied, therefore, this review broadly summarizes previous research to provide clues for future basic and clinical studies.

## Identification of C-Kit^+^ Cells in the Liver

Several cell surface markers have been utilized to identify stem/progenitor cells of the liver, such as c-kit, CD34, Thy-1 (Lemmer et al., 1998; Petersen et al., 1998; Baumann et al., 1999; Crosby et al., 2001). Even without a characteristic marker for liver progenitor cells, previous studies used c-kit as a potential marker for liver stem/progenitor cells (Fujio et al., 1994; Fujio et al., 1996; Baumann et al., 1999). Adult hepatic stem cells, also termed oval cells, express both SCF and c-kit (Fujio et al., 1994). While the proliferation of hepatocytes is inhibited, KIT-mediated signal transduction activates oval cells which then produce hepatocytes and bile epithelial cells (BECs) (Matsusaka et al., 1999). Besides, c-kit is expressed in oval cells and BECs (Fujio et al., 1996), whereas only 20% of hepatocytes located mainly at the periportal and pericentral regions express c-kit with a weak staining intensity (Yushkov et al., 2011). Moreover, liver sinusoidal endothelial cells (LSECs) are known to express c-kit (Luna et al., 2004). On the other hand, c-kit can be detected in early hematopoietic cells, mast cells, and dendritic cells. And c-kit^+^ mast cells are closely related to primary sclerosing cholangitis (PSC) (Metcalfe et al., 1997; Ishii et al., 2005; Ray et al., 2010). Kupffer cells and hepatic stellate cells are negative for c-kit (Ikarashi et al., 2013; Fu et al., 2015).

## C-Kit and Liver Regeneration

**FIGURE 1:**
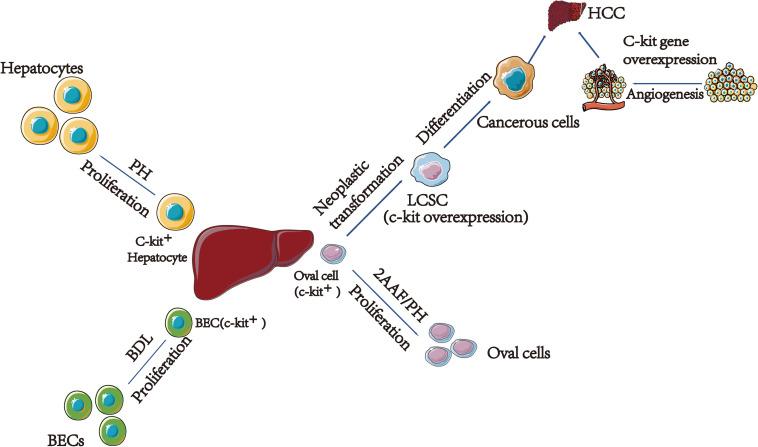
The relationship between c-kit and different type of cells in liver regeneration and cancer. (1) C-kit express in 20% hepatocytes. After acute injury, solubilized SCF released and interact with c-kit, leading surrounding hepatocytes prolifer8ation. (2) In young rats after BDL, SCF bind to c-kit, leading to activation of the MAPK pathway; finally, BECs proliferation. (3) C-kit play a vital role in HCC. On the one hand, oval cells transformed to LCSCs are related with the c-kit gene overexpression. On the other hand, c-kit might associate with HCC by participating in angiogenesis. PH, Partial Hepatectomy; BEC, Bile Epithelial Cell; BDL, Bile Duct Ligation; 2AAF/PH, 2-Acetylaminofluorene/Partial Hepatectomy; LCSC, Liver Cancer Stem Cell; HCC, Hepatocellular Carcinoma.

The liver is the only visceral organ with the capacity to regenerate. Liver c-kit^+^ cells, including part of hepatocytes, BECs, and oval cells participate in liver regeneration by differentiating into different types of cells depending on the type of the injury. Moreover, c-kit^+^ hematopoietic stem/progenitor cells (HSCs/HPCs) participate in liver regeneration. SCF/KIT signal transduction system regulates cell proliferation and survival. This review explains the relationship between c-kit and liver regeneration based on different types of cells (Figure 1).

### Hepatocytes

Stem cell growth factor is localized in hepatocytes surrounding the BECs. And c-kit expressed with a weak staining intensity in 20% of hepatocytes located mainly at the periportal and pericentral regions (Yushkov et al., 2011).

**FIGURE 2:**
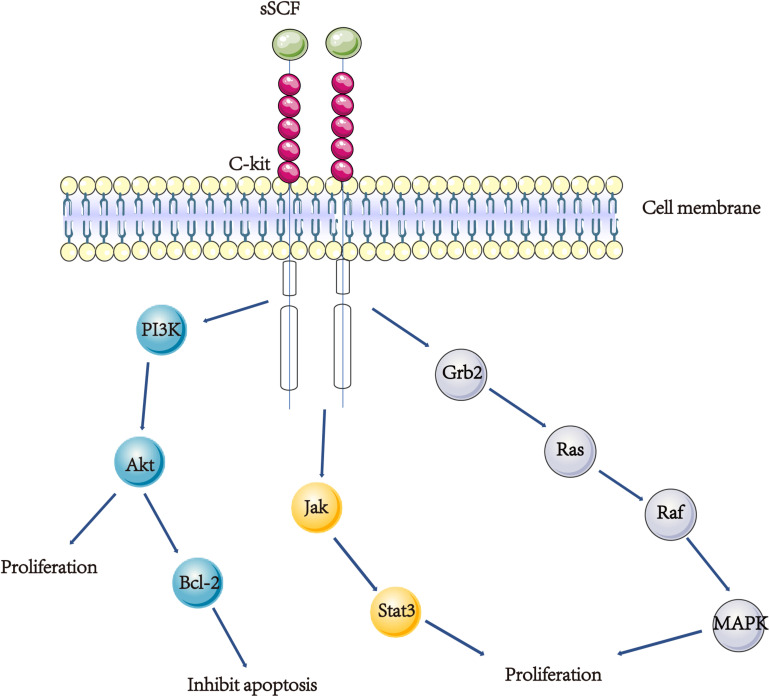
A scheme showing the SCF/KIT signal transduction. (1) SCF/KIT function in the APAP model might be mainly through increasing proliferation of hepatocyte and inhibiting hepatocyte apoptosis. Exogenous SCF activating c-kit and Akt, results in an increase in Bcl-2 protein and inhibiting hepatocyte apoptosis. (2) IL-6 stimulates the production and release of SCF, then SCF interacts with c-kit in activating the stat3 signal pathway thereby inducing hepatocyte proliferation. (3) Both SCF and c-kit are up-regulated in BECs in young rats after BDL, SCF binds to c-kit, causing the activation of the Ras/Raf/MAPK pathway, hence the proliferation of bile epithelial cells.

Partial hepatectomy (PH) is the most common model used to study liver regeneration. After PH, mature hepatocytes proliferation leads to liver regeneration. Following PH, there is a significant decline in liver SCF levels and an increase in serum SCF levels during the first 24 h (Ren et al., 2003). The level of liver SCF mRNA was upregulated from the 24th hour to the 7th day (Meng et al., 2012). The number of c-kit^+^ hepatocytes increased after PH, including medium and high staining intensity (Yushkov et al., 2011). In addition, exogenous SCF promotes the proliferation of primary hepatocytes in humans and mice (Meng et al., 2012). Moreover, a study by Ren et al. linked the interleukin-6 (IL-6) enzyme and tumor necrosis factor-α (TNF-α) with the SCF/C-kit in mice in 70% PH model. Results suggested that IL-6 stimulates the production and release of SCF, then SCF interacts with c-kit in activating the signal transducer and activator of transcription 3 (stat3) signal pathway thereby inducing hepatocyte proliferation (Ren et al., 2003; Figure 2). Besides, TNF-α-induced hepatocyte proliferation was partially mediated via SCF/c-kit, and the SCF-induced hepatocyte proliferation might also be mediated by the stat3 signal pathway (Ren et al., 2008; Figure 2). The possible mechanism occurs after acute injury where solubilized SCF is released and interact with c-kit, causing proliferation of the surrounding hepatocytes.

Acetaminophen or Tylenol overdose has become the most prevalent cause of acute liver failure in the United States (Larson et al., 2005). Overdose produces serious acute liver toxicity, leading to hepatocyte necrosis, acute liver failure, and even death. Acetaminophen (APAP) model is a widely used animal experimental model of drug-induced hepatic injury. Data from an examination by Hu and Colletti et al. on the c-kit and SCF expression of C57BL/6J mice, shows that c-kit mRNA increase begins at 6 h and peaks after 48 h of overdose APAP treated, while SCF mRNA increase begins at 6 h and peaks at 16 h post-administration. Treatment with exogenous SCF significantly reduces the mortality of mice in 750 mg/kg APAP model. These findings suggest that SCF/KIT’s function in the APAP model might be mainly through increasing proliferation of hepatocyte and inhibiting hepatocyte apoptosis. Exogenous SCF activating c-kit and Akt, results in an increase in B cell lymphoma-2 (Bcl-2) protein and inhibiting hepatocyte apoptosis (Hu and Colletti, 2008; Figure 2). In contrast, inhibiting c-kit will increase the mortality of the lethal dose APAP-treated model (Nassar et al., 2009; Shaker, 2014). In summary, these results indicate that c-kit is an important molecule in liver recovery according to the APAP model. Activation of the SCF/KIT system causes an increase in hepatocyte proliferation and a decrease in hepatocyte apoptosis.

### Bile Epithelial Cells

Studies argue that the proliferation of BEC is associated with c-kit. A study by Fujio et al. (1996) reported that SCF and c-kit are co-expressed in BEC during the embryonic and adult stages of rat’s liver. Elsewhere, Omori et al. demonstrated that BECs in young liver react differently with adult liver in the bile duct ligation (BDL) model. In the liver of a young rat with 5 weeks of age, there is an upregulation in the expression of SCF and c-kit after BDL is performed. But in the adult liver of rats after BDL, no upregulation of SCF and c-kit is observed. And the pattern of BECs in young liver response to bile stasis is similar to that of the early stage of oval cell activation (Omori et al., 1997). Evidence reported by Satake et al. show that both SCF and c-kit are up-regulated in BECs in young rats after BDL, SCF binds to c-kit, causing the activation of the Ras/Raf/mitogen-activated protein kinase (MAPK) pathway, hence the proliferation of bile epithelial cells (Figure 2). Nevertheless, in the adult rats, KIT-mediated signal transduction plays a mirror role due to the low expression of SCF/c-kit (Satake et al., 2003).

### Oval Cells

Oval cells are the progenitor cells of the liver with c-kit as one of its markers (Crosby et al., 2001). Normally, after PH, mature hepatocytes proliferate causing liver regeneration. However, if PH is performed when the replicative ability of mature hepatocytes is impaired, there will occur proliferation and differentiation of oval cells (Thorgeirsson, 1996). PH integrated with 2-acetylaminofluorene (2-AAF)/PH is the model utilized to investigate the liver regeneration capability via oval cells. Further reports by Fujio et al. (1994) showed that in the embryonic and adult rats, both SCF and c-kit were expressed in oval cells. After 2-AAF/PH, the level of SCF transcripts increased within 12 h and reached a peak at day 4, the level of c-kit mRNA was upregulated from 12th hour to 11th day (Fujio et al., 1994). Additionally, Matsusaka et al. used Ws/Ws rats with 2-AAF/PH model to evaluate the role of SCF/KIT in the development of oval cells. Their results demonstrated that in at least one rat under the 2-AAF/PH model, KIT-mediated signal transduction activates the oval cells. However, in the proliferative activity and determining the phenotype of oval cells, the SCF/KIT signal transduction system plays a minor role (Matsusaka et al., 1999).

### Circulating Progenitor Cells

**FIGURE 3:**
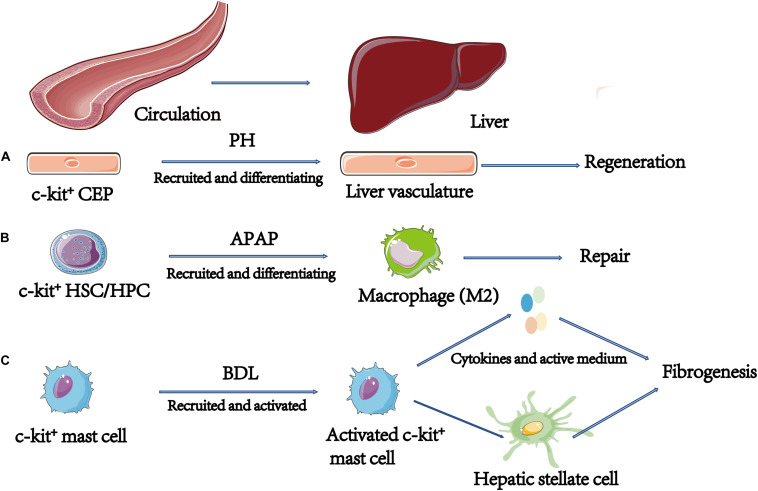
The role of c-kit^+^ circulating progenitor cells in liver diseases. **(A)** By combined bone marrow transplant with 70% PH, the investigators conclude that CEPs contribute to liver regeneration by differentiating into liver vasculature. **(B)** In APAP model, c-kit^+^ HSCs/HPCs were recruited into liver and these HSCs/HPCs express the macrophage M2 (repair phenotype) genes characteristic. **(C)** Cholestatic/biliary diseases-associated fibrosis. Damaged BECs express and secrete SCF to recruit mast cells, then mast cells migrate to the damage area and contribute to fibrogenesis. C-kit^+^ mast cells play a key role in fibrogenesis mainly through expressing fibrosis-associated factors including tryptase, b-FGF and TNF-α. HSCs/HPCs, Hematopoietic Stem/Progenitor Cells; CEPs, Circulating Endothelial Progenitors.

Circulating endothelial progenitors (CEPs) originated from bone marrow with markers c-kit or CD133. By combining bone marrow transplant with 70% PH, the investigators concluded that CEPs contribute to liver regeneration by differentiating into liver vasculature, and exogenous VEGF accelerates liver regeneration by increasing the recruitment of CEPs in 70% PH model (Beaudry et al., 2007; Figure 3A). Moreover, a study by Si et al. found that there were relatively more CD45^+^Lin^–^C-Kit^+^ HSCs/HPCs recruited in wide type (WT) liver than chemokine (C-C motif) receptor2 knock out (CCR2^–/–^) after APAP-induced injury. More importantly, after administration with exogenous CD45^+^Lin^–^C-Kit^+^ HSCs/HPCs, WT had greater resolution of APAP-induced injury than CCR2^–/–^ mice, and these HSCs/HPCs express the macrophage M2 (repair phenotype) genes characteristic (Si et al., 2010; Figure 3B).

In summary, liver regeneration is a complex process, with multiple mechanisms and pathways, current literatures suggest that c-kit regulates liver regeneration. On the one hand, activation of the SCF/KIT signal transduction system leads to hepatocytes proliferation and apoptosis inhibition, BECs proliferation and oval cells activation. On the other hand, c-kit^+^ progenitor cells contribute to liver regeneration by differentiating into liver vasculature or alternatively activated macrophages.

## C-Kit and Liver Diseases

C-kit is expressed on HSCs/HPCs and mast cells, and several liver diseases are relevant to c-kit. A strong relationship was observed between c-kit and primary liver cancer, besides, c-kit^+^ mast cells also participate in fibrogenesis. This section extensively summarizes the relationship.

### Primary Liver Cancer

Liver cancer is the fourth leading cause of cancer death among sexes combined globally (Bray et al., 2018). Evidence shows that liver stem/progenitor cells are the potential source of HCC, also called liver cancer stem cells (LCSCs) (Dumble et al., 2002; Fujii et al., 2008). It is believed that the liver stem cells transformed into LCSCs are linked with the overexpression of the c-kit gene (Chen et al., 2007; Yan et al., 2018). The SCF/c-kit signal transduction system participates in the activation and proliferation of oval cells. On the other hand, overexpression of c-kit is relevant with micro-vessel density, therefore, c-kit might be associated with HCC by participating in angiogenesis (Yan et al., 2018). Concerning the hepatitis B virus (HBV) or hepatitis C virus (HCV) induced HCC, c-kit is involved. PreS1 protein of HBV stimulates the appearance and self-renewal of LCSCs by activating the expression of c-kit and is confirmed in human hepatoma cells and HCC tissues (Liu et al., 2017). And in HCV infection patients, c-kit overexpression is observed (Kwon et al., 2015; Nazzal et al., 2020). Data shows that HCV core protein upregulates the expression of c-kit in hepatocytes, inducing the epithelial-mesenchymal transition, and extends the life span of cells (Kwon et al., 2015). Further, evidence recommends that liver stem/progenitor cells are the potential source of intrahepatic cholangiocarcinoma (ICC) and SCF/c-kit system might contribute to ICC by promoting cell proliferation and survival (Nomoto et al., 2006; Komuta et al., 2008; Mansuroglu et al., 2009b). Again, several lines of evidence attest that liver stem/progenitor cells are the potential source of HCC-ICC (Tanaka et al., 2005; Zhang et al., 2008). And the recent classification of digestive system tumor (5th edition) by the World Health Organization (WHO) also agrees with this concept. A different follow-up model indicates that the recurrence of HCC-ICC after hepatectomy is associated with the expression of c-kit in both tumor and non-tumor livers (Cai et al., 2012).

HCC is the most prevalent primary liver cancer, accounting for 85∼90% of primary liver cancers. However, the positive rate of HCC has not been precisely clarified. A research executed by Chung et al. (2005) examined the protein expression of c-kit in 86 human HCC specimens. The results demonstrated that among the 86 specimens, 22 (25.6%) were positive. According to an article published by Yan et al. (2018), the percentage of positive c-kit expression is 48.1% in 206 HCC cases. Nevertheless, Becker et al. (2007) collected 258 human HCC samples and c-kit immunohistochemistry was performed, results reported that only 6 (2.3%) samples were positive. There are many reasons attributed to these results among them, antibody specificity (Went et al., 2004), cellular heterogeneity, different stages of cell differentiation, etc. In summary, more cases need to be included and more detailed etiology should be classified. Epidemiological data shows that ICC accounts for 15% of primary liver cancer, second to the HCC (Shaib et al., 2005). Clinicopathological study on c-kit positive immunohistochemistry staining of human ICC sample (1/32), suggesting that ICC originates from hepatic stem cell (Liu et al., 2004). Certainly, only one c-kit positive among 32 samples could not clarify the origin of ICC, implying that other mechanisms might also exist. Therefore, more cases need to be collected in future studies before a single-cell RNA sequence can be performed, this might solve the problem of origin. A study by Xu et al. (2018) showed that NCAM^+^ c-kit^+^ subset cells in RBE cell lines might have properties of hepatic progenitor cells. And NCAM combined with c-Kit might be a valuable marker for isolating and purifying ICC stem/progenitor cells. Therefore, c-kit can be a candidate marker of ICC stem/progenitor cells used in single cell RNA sequence in the future. HCC-ICC is a rare subtype of primary liver cancer with poor prognosis, comprising <1% of all liver carcinomas, and epidemiological data shows it accounts for 1.6–6.5% of surgically resected primary liver cancer (Koh et al., 2005; Yu et al., 2011; Akiba et al., 2013). Unlike the low c-kit positive rate of HCC, the presence of the c-kit in HCC-ICC is 71.4% (10/14), which might explain the poor prognosis of HCC-ICC (Yu et al., 2011).

In addition, it is reported that there is positive feedback in transforming growth factor β (TGF-β) and c-kit, which induce carcinogenesis in HepG2 cells. TGF-β activated SMAD2 transcriptionally induces SCF expression, SCF expression and secretion result in stimulation of the c-kit receptor, followed by activation of JAK1/STAT3 signaling. p^*Tyr*705^STAT3 trans-locates to the nucleus where it binds directly to the TGF-β ligand gene, positively regulating its expression. Following activation of secreted TGF-β precursor, it can activate the TGF-β receptor. Disruption of the TGF-β/KIT signaling loop on the level of the SCF/STAT3 axis restores TGF-β tumor suppressor function by inhibition of epithelial-mesenchymal transition (EMT), tumor cell migration, and invasion, and restoration of its cell cycle inhibitory functions (Rojas et al., 2016). Besides, a recent study showed that in HCV-infected patients, their c-kit gene mutation in exons 9 but not 11, are often bound up with high- viremia HCV. And these patients might be resistant to TGF-β, thus promoting the development of HCC (El-Houseini et al., 2019). These studies indicate that TGF-β/KIT signaling plays an important role in the development of HCC and is a key target for the prevention and treatment of HCC.

### Hepatic Fibrosis

Hepatic fibrosis is a wound healing process involving multiple cell types. There are many causes of liver fibrosis, including viral, alcoholic, cholestasis, etc. The role of c-kit in liver fibrosis is quite complex. Mast cells increase in liver fibrosis, but decrease in other chronic liver diseases, suggesting that mast cells take part in fibrogenesis particularly in cholestatic/biliary diseases (Weiskirchen et al., 2019).

#### Cholestatic/Biliary Diseases-Associated Fibrosis

Most of hematopoietic cells lose expression of c-kit when they mature, except mast cells and dendritic cells. Mast cells are derived from the bone marrow, and they act as immature progenitors in circulation. In the liver, mast cells are primarily located in the periportal area. Primary sclerosing cholangitis (PSC) or primary biliary cirrhosis is an autoimmune disease characterized by infiltration of mast cell (Nakamura et al., 1997; Ishii et al., 2005). In contrast, there is no significant increase in acute viral or drug-induced hepatitis (Yamashiro et al., 1998).

PSC is a disease characterized by infiltration of mast cell, biliary damage, and liver fibrosis. SCF/KIT signaling plays a critical role in the development of mast cell, survival and homing. Animal models have shown that inhibiting the c-kit system releases allergic reactions in the lungs (Berlin et al., 2004). In the liver, data suggests that c-kit^+^ mast cells play a key role in fibrogenesis from the early stage of PSC (Ishii et al., 2005; Meadows et al., 2019). First, damaged BECs express and secrete SCF to recruit mast cells. Secondly, mast cells migrate to the damaged area and cause fibrogenesis. Therefore, c-kit^+^ mast cells contribute to fibrogenesis primarily through expressing fibrosis-associated factors including tryptase, basic fibroblast growth factor (b-FGF), and TNF-α (Kanbe et al., 2000; Gaca et al., 2002; Figure 3C). BDL-induced biliary hyperplasia, hepatic injury, and fibrosis are reduced in Kit^*W–sh*^ mice, which are mast cell deficient (Hargrove et al., 2017). As for ameliorating progression of PSC, targeting mast cell infiltration might be an efficient option. Furthermore, in systemic mastocytosis, mastocytosis-derived extracellular vesicles transfer c-kit to liver stellate cells, causing activation, proliferation, cytokine production, and differentiation of liver stellate cells (Kim et al., 2018). This might be an alternative mechanism of c-kit^+^ mast cells-induced fibrogenesis.

Additionally, mast cells take part in the progress of biliary atresia (BA). It is reported that the increased mast cells adversely affects liver function, perhaps through type I allergic reaction (Uddin Ahmed et al., 2000). However, there is no specific study about the SCF/c-kit system and BA. Given the relationship between mast cells and BA, SCF/c-kit system should be considered.

#### Other Chronic Liver Diseases-Associated Fibrosis

The number of mast cells in other chronic liver diseases-associated fibrosis is increased, and the intensity of c-kit immunostaining is slightly higher in cirrhotic non-tumorous liver than in non-cirrhotic non-tumorous liver, but the relationship between c-kit and fibrosis has not been extensively evaluated (Mansuroglu et al., 2009a). C-kit expressed in fibroblasts and SCF/c-kit plays a vital role in scar pathogenesis, thus we can use a c-kit selective inhibitor to block it (Mukhopadhyay et al., 2011).

In summary, the role of c-kit in liver fibrosis is obscure. In cholestatic/biliary diseases-associated fibrosis, c-kit^+^ mast cells regulate fibrogenesis. However, in other chronic liver diseases-associated fibrosis, despite the increase of mast cells, the relationship between c-kit and fibrosis is largely understudied. Therefore, further studies are necessary to elaborate on the relationship between c-kit and hepatic fibrosis.

### Other Liver Diseases

The roles of c-kit^+^ cells in chronic hepatitis B and C have been described in HCC (Kara et al., 2008; Kwon et al., 2015; Liu et al., 2017; Nazzal et al., 2020). Also, it is reported that there is an increase of mast cells in alcoholic hepatitis, but reports on the relationship between c-kit and alcoholic hepatitis are insufficient (Farrell et al., 1995). Alcoholic hepatitis impairs intestinal barrier and activate the mast cell causing fibrogenesis (Ferrier et al., 2006). Besides, a study by Hisada et al. (2017) mentioned that the percentage of c-kit^+^ cells was dramatically decreased in alcohol-fed rats compared to non-alcohol-fed rats. These findings indicate that BMSCs might be damaged by the consumption of alcohol. Nonetheless, the relationship between c-kit^+^ cells and alcohol has not been fully elucidated, hence this represents an important topic for future research.

In summary, c-kit is relevant to primary liver cancer. It is believed that liver stem cells transformed into LCSCs are linked with the overexpression of the c-kit gene, causing liver cancer. Besides, c-kit^+^ mast cells participate in fibrogenesis particularly in cholestatic/biliary diseases. C-kit^+^ mast cells contribute to fibrogenesis primarily through expressing fibrosis-associated factors.

## Clinical Implications of C-Kit in Liver

### The Role of C-Kit in Diagnosis and Prognosis

Few reports are suggested that c-kit can be used as a diagnostic factor in liver diseases. For instance, Kara et al. (2008) recommended that c-kit can be used as an early diagnostic factor for HBV-related HCC. However, it is unclear whether c-kit can be used as an indicator in HCC caused by other factors. Furthermore, it is reported that c-kit^+^ mast cells increase after liver allograft rejection (El-Refaie and Burt, 2005), but the increased c-kit^+^ mast cells cannot distinguish rejection from recurrent HCV infection in transplantation of liver (Doria et al., 2006). Seemingly, c-kit is a good prognostic parameter in several diseases. First, one article has mentioned that c-kit can be used as a prognostic factor for HCC (Chung et al., 2005). Moreover, Yan et al. suggested that c-kit is an independent prognostic indicator for HBV-related HCC patients. In addition, Kaplan–Meier survival analysis shows that the c-kit expression was linked to poor disease-free survival (DFS) (*P* < 0.001) in HBV-related HCC patients (Yan et al., 2018). Besides, in a cohort of 70 HCC-ICC patients who underwent resection for treatment, overall survival (OS) and DFS were associated with expression of c-kit in both tumor and non-tumor livers (Cai et al., 2012). Secondly, the increased number of c-kit^+^ mast cells in chronic HCV patients might be used as an indicator of liver fibrosis (Koruk et al., 2011). Thirdly, it is reported that the number of mast cells adversely affects liver function in biliary atresia, but the authors did not stain mast cells with c-kit. Therefore, more patients should be enrolled when conducting c-kit^+^ mast cell statistics (Uddin Ahmed et al., 2000). Finally, due to the role of c-kit^+^ mast cells in fibrogenesis among PSC patients and animal models, the number of c-kit^+^ mast cells might reflect the prognosis of PSC patients (Ishii et al., 2005; Meadows et al., 2019). However, only 4 samples were observed, therefore more clinical data need to be counted.

### The Role of C-Kit^+^ Cell in Stem Cell Therapy

Oval cell transplantation after liver transplantation might be useful for liver regeneration. A study by Li et al. (2013) isolated oval cells, of which 99.8% were positive for c-kit. After primary culture, oval cells were injected into the rats who underwent liver transplantation. Data shows that compared to controls, oval cell transplantation significantly improved the liver function, prolonged median survival time and reduced acute allograft rejection (Li et al., 2013).

**FIGURE 4:**
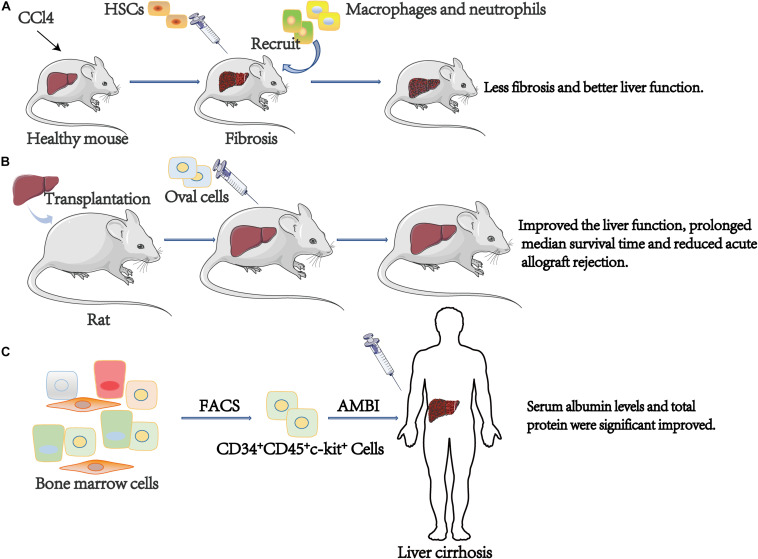
The role of c-kit^+^ cell in stem cell therapy. **(A)** CCl4 treated liver fibrosis mice administered with the HSCs (Lin^–^Sca-1^+^c-kit^+^) had less fibrosis and better liver function compared with the group not inject. **(B)** Compare to controls, oval cells transplantation significantly improved the liver function, prolonged median survival time and reduced acute allograft rejection. **(C)** After infusing, significantly improved serum albumin levels and total protein were observed. ABMI, Autologous Bone Marrow cell Infusion.

BMSCs are closely related to liver fibrosis. In carbon tetrachloride (CCl4)-induced liver fibrosis mice, the differentiation potential of circulation of endothelial progenitor cells (EPCs) is attenuated due to the impairment of the endothelial lineage differentiation potential of bone marrow-C-kit^+^, Sca-1^+^, and Lin^–^(BM-KSL) cells (Shirakura et al., 2011). Transplantation of HSCs has been reported to be effective in mice. Some authors proved that CCl4 treated liver fibrosis mice administered with the HSCs (Lin^–^Sca-1^+^c-kit^+^) had less fibrosis and better liver function compared with the group not inject (Cho et al., 2011). Moreover, King et al. arrived at the same conclusion and pointed out that HSCs (Lin^–^Sca-1^+^c-kit^+^) participate in anti-fibrotic by promoting the recruitment of endogenous macrophages and neutrophils. And the retention of HSCs can be increased by reducing sphingosine-1-phosphate (S1P) signaling to antifibrosis (King et al., 2017). Furthermore, it has been reported that in CCl4-induced liver injury, transplantation of very small embryonic-like stem cells (VSELs), which express c-kit, directly into the liver can significantly lower the levels of serum alanine aminotransferase (ALT) and aspartate aminotransferase (AST) (Chen et al., 2015). Transplantation of BMSCs has been reported to be effective in humans. Research by Terai et al. reported 9 patients with liver cirrhosis that received the therapy of autologous bone marrow cell infusion (ABMI). The cell characteristics were confirmed by fluorescence-activated cell sorting analysis (CD34^+^, CD45^+^, c-kit^+^). And after infusing, significantly improved serum albumin levels and total protein were witnessed. Besides, significant improvements in Child-Pugh scores were observed (Terai et al., 2006; Figure 4).

By and large, we can speculate that c-kit^+^ oval cell transplantation might be useful in therapeutic liver regeneration after orthotopic liver transplantation. And c-kit^+^ BMSCs or HSCs transplantation is a promising treatment of patients with liver cirrhosis. Nevertheless, considering the role of c-kit in liver cancer, further studies are essential to evaluate the relationship between c-kit^+^ cell, hepatic fibrosis, and liver cancer. Additionally, we summarized the clinical implications of c-kit in liver (Table 1).

**TABLE 1:** Clinical implications of c-kit in liver.

Clinical implications				References
**Diagnosis/prognosis**	**Conclusions**			
	C-kit can be used as an early diagnostic factor for HBV-related HCC.			Kara et al., 2008
	C-kit^+^ mast cells increase after liver allograft rejection, but the increased c-kit^+^ mast cells cannot distinguish rejection from recurrent HCV infection in transplantation of liver.			El-Refaie and Burt, 2005; Doria et al., 2006
	C-kit can be used as a prognostic factor for HCC. Moreover, c-kit is an independent prognostic indicator for HBV-related HCC patients.			Chung et al., 2005; Cai et al., 2012; Yan et al., 2018
	The increased number of c-kit^+^ mast cells in chronic HCV patients might be used as an indicator of liver fibrosis.			Koruk et al., 2011
	The number of c-kit^+^ mast cells might reflect the prognosis of PSC patients.			Ishii et al., 2005; Meadows et al., 2019

**Treatment**	**Species/model**	**Stem cell therapy**	**Results**	

	Rats/underwent liver transplantation	Oval cells (99.8% c-kit^+^)	Significantly improved the liver function, prolonged median survival time and reduced acute allograft rejection	Li et al., 2013
	Mice/CCl4 induced liver fibrosis	HSCs (Lin^–^Sca-1^+^c-kit^+^)	Less fibrosis and better liver function compared with the group not inject	Cho et al., 2011
	Mice/CCl4 induced liver fibrosis	HSCs (Lin^–^Sca-1^+^c-kit^+^)	Participate in anti-fibrotic by promoting the recruitment of endogenous macrophages and neutrophils.	King et al., 2017
	Mice/CCl4-induced liver injured	VSELs (Lin^–^Sca-1^+^c-kit^+^CD45^–^)	Lower the levels of ALT and AST	Chen et al., 2015
	Humans/Liver cirrhosis	Bone marrow cell (CD34^+^, CD45^+^, c-kit^+^)	Significantly improved serum albumin levels and total protein	Terai et al., 2006

### Targeted Inhibition of C-Kit in Liver Diseases

Besides its implication in prognosis and stem cell therapy, overexpression of c-kit in liver primary cancer has potential therapeutic implications because it can inhibit the kinase activity of c-kit. Imatinib mesylate (STI-571 or Gleevecs) is a relatively selective tyrosine inhibitor of BCR-ABL, PDGF-R, and c-kit, which is usually used in the treatment of chronic myelocytic leukemia (CML) and GIST. Animal experiments point out that imatinib treatment can inhibit c-kit-expressing liver progenitor cells proliferation and early fibrosis induced by short-term choline-deficient, ethionine-supplemented (CDE) diet. In the long run, imatinib treatment can reduce the degree of fibrosis and significantly inhibit the formation of tumor (Knight et al., 2008). Furthermore, Nazzal et al. recently showed that HCC-patient-derived xenograft (PDX) tumors respond well following imatinib treatment. Treatment of HCC-PDX xenograft tumor-bearing mice with imatinib significantly reduced tumor growth and c-kit downstream molecules expression (Nazzal et al., 2020). In clinical, Becker et al. (2007) discovered that the positive rate of c-kit is 2.3% and that there was no role for imatinib. Similarly, in a clinical phase II trial of imatinib, Eckel et al. (2005) found no positive case for c-kit among 15 HCC patients, and imatinib showed no therapeutic effect. Another phase II study of imatinib in unresectable HCC concluded that imatinib is unsuitable as a monotherapy in the treatment of unresectable HCC (Lin et al., 2008). Interestingly, Ramadori et al. (2004) effectively treated one HCC patient with imatinib. Sorafenib is a multi-kinase inhibitor that is broadly applied in unresectable HCC as a first-line treatment. C-kit is among the molecular targets of sorafenib. In one case report, a patient diagnosed with HCC-ICC and highly c-kit positive, responded to sorafenib (Seino et al., 2014). As we know, sorafenib targets c-kit, and the high concentration of soluble c-kit group has a greater response to sorafenib than the low concentration group (Llovet et al., 2012). Since primary liver tumors are regulated by multiple signaling pathways, imatinib is hardly used in patients with HCC. But going by the results of sorafenib and imatinib, the c-kit pathway should be of concern. Additionally, we summarized c-kit signal involved drugs and clinical trials in unresectable hepatocellular carcinoma(uHCC)(Table 2).

**TABLE 2:** C-kit signal involved drugs and clinical trials in unresectable hepatocellular carcinoma.

Drug	Targets	Trial for uHCC		References
		Status	Phase	
Sorafenib	VEGFR1-3, PDGFR-β, c-KIT, RET, FLT-3, RAF	First-line treatment		Llovet et al., 2008; Cheng et al., 2009; Pressiani et al., 2013
Lenvatinib	VEGFR1-3, FGFR1-4, c-kit, RET, PDGF-β	First-line treatment		Kudo et al., 2018
Regorafenib	VEGFR1-3, c-Kit, TIE-2, PDGFR-β, FGFR-1, RAF-1, BRAF, p38	Second-line treatment		Bruix et al., 2017
Anlotinib	VEGFR2/3, FGFR1-4, PDGFRα/β, c-kit, Ret	Ongoing	II	Clinicaltrials.gov
Sunitinib	PDGFR, VEGFR1-2, c-kit, FLT3, RET	Unsuccessful	II	Faivre et al., 2009
Dovitinib	FGFR1&3, VEGFR1-3, PDGFR-β, c-kit	Unsuccessful	II	Cheng et al., 2016
Axitinib	VEGFR1-3, c-kit, PDGFR	Unsuccessful	II	Kang et al., 2015
Imatinib	Bcr-Abl, PDGFR, c-kit	Unsuccessful	II	Lin et al., 2008

On the other hand, due to the role of c-kit^+^ mast cells in cholestatic/biliary diseases-associated fibrosis, c-kit receptor tyrosine inhibitor can be used to prevent fibrosis. It is reported that a c-kit/PDGF receptor tyrosine kinase inhibitor-masitinib, plays an effective role in the treatment of corticosteroid-dependent asthma by inhibiting c-kit^+^ mast cells (Humbert et al., 2009). However, to validate these findings, further *in vitro* and *in vivo* experiments are essential.

## Summary and Future Perspectives

The role of c-kit in the liver is conflicting, it is a two-edged sword in liver regeneration and diseases. First, as a liver stem cell marker, c-kit^+^ cells, such as oval cells, BECs, and part of hepatocytes are closely linked to liver regeneration. And c-kit signaling participates in cell proliferation, migration, and survival based on different types of cells. At the final stage of chronic liver cirrhosis, liver transplantation is the most effective treatment. But there are many challenges such as limited donors, surgical complications, etc. And c-kit^+^ BMSCs or HSCs transplantation is a potential treatment of patients in the final stage of chronic liver cirrhosis. Nevertheless, further research is crucial to explore the relationship between c-kit^+^ cell, hepatic fibrosis, and liver cancer. Secondly, mast cells express c-kit, and damaged BECs express and secrete SCF to recruit c-kit^+^ mast cells, resulting in fibrogenesis. Eventually, c-kit also is a proto-oncogene, and its overexpression is associated with primary liver cancer. Sorafenib, a multi-kinase inhibitor with c-kit included, is used as a first-line treatment for HCC. Since primary liver tumors are regulated by multiple signaling pathways, imatinib is hardly used in patients with HCC. But going by the results of sorafenib and imatinib, the c-kit pathway should be of concern.

Conventional research methods cannot reveal the dynamic process of stem cell differentiation *in vivo*, but genetic lineage tracing can address this challenge. Previously, we used Kit-CreERxRosa26-RFP mouse to trace the role of c-kit^+^ stem/progenitor cells in vascular injury-induced neointimal lesions, and found that c-kit^+^ cells are majorly converted to immune-inflammatory cells during neointima formation (Chen et al., 2018). Another laboratory utilized Kit^*MerCreMer/+*^x Rosa26-eGFP lineage-tracing mouse to examine endothelial cell formation in the heart (Vagnozzi et al., 2020). Furthermore, several laboratories used single-cell RNA sequences to reveal the cell heterogeneity of the liver, including all liver cells, immune cells, and cancer stem cells (Zheng et al., 2018; Aizarani et al., 2019; Zhang et al., 2019). Recently, lineage-tracing combine RNA sequence analysis has revealed the mechanisms of endothelial repair by progenitors (Deng et al., 2020). In future research, this technology can be adopted in the liver to expose the role of c-kit^+^ cells in various liver diseases.

## Author Contributions

WW wrote the manuscript and prepared figures. MZ, YL, and LS provided the expert comments and edited the manuscript. All authors reviewed the manuscript.

## Conflict of Interest

The authors declare that the research was conducted in the absence of any commercial or financial relationships that could be construed as a potential conflict of interest.
